# Epidemiological and clinical analysis of *Gelsemium* poisoning in Guangxi, Southern China: a decade of surveillance data

**DOI:** 10.3389/ftox.2026.1821880

**Published:** 2026-07-02

**Authors:** Yanxu Zhong, Mengmeng Shi, Wei Mao, Huitian Deng, Xiaofeng Huang, Yuyan Jiang

**Affiliations:** 1 Guangxi Zhuang Autonomous Region Centre for Disease Control and Prevention, Nanning, China; 2 Guangxi Key Laboratory for the Prevention and Control of Viral Hepatitis, Guangxi Zhuang Autonomous Region Centre for Disease Control and Prevention, Nanning, China; 3 Guangxi Zhuang Autonomous Region Academy of Preventive Medicine, Nanning, China; 4 School of Public Health, Guangxi Medical University, Nanning, China; 5 School of Public Health, Youjiang Medical University for Nationalities, Baise, China

**Keywords:** clinical features, epidemiology, gelsemium, poisoning, surveillance

## Abstract

**Background:**

*Gelsemium elegans* Benth, commonly known as “duan chang cao”, is a highly toxic plant belonging to the family *Gelsemiaceae*. It is distributed in southern China with its potent toxicity poses a persistent threat to public health.

**Objective:**

This study aims to comprehensively characterize the epidemiology and clinical features of *Gelsemium* poisoning in Guangxi, Southern China, based on surveillance data, in order to provide an evidence-based foundation for timely and effective prevention and control measures.

**Methods:**

A secondary analysis was conducted on 10 years of *Gelsemium* poisoning surveillance data (2015–2024) from Guangxi. Descriptive statistics were applied to summarize epidemiological patterns and clinical manifestations.

**Result:**

From 2015 to 2024, *Gelsemium* poisoning in Guangxi showed a high but fluctuating mortality rate, peaking at 100% in 2015. The highest mortality risk (19.0%) occurred in individuals aged 20–59 years. Cases clustered geographically in Chongzuo, Yulin, Guigang, Qinzhou, and Laibin, with incidence peaking in autumn. Alcohol infusion was the leading cause of fatal poisoning (29.5%), mainly in household and rural banquet settings. Consumption of the rhizome resulted in the highest death rate (21.1%). Dizziness and blurred vision were common clinical symptoms. Significant differences in incidence, hospitalization, and mortality rates were observed based on location, season, cooking method, source part, and misidentification (*P* < 0.05).

**Conclusion:**

Enhancing surveillance, food safety regulations, and targeted health education plays a key role in preventing *Gelsemium* poisoning. Establishing strong monitoring and quick intervention systems may further reduce its impact.

## 1 Introduction

*Gelsemium*, commonly known as “Gou-Wen” or “Duan-Chang-Cao” in China, is an evergreen woody vine of *Gelsemiaceae* family. The genus comprises three species: *Gelsemium elegans* Benth., native to Southeast Asia, and *Gelsemium sempervirens* Ait. and *Gelsemium rankinii* Small., both found in North America. *Gelsemium elegans* is widely distributed in southern China and has been traditionally used for its medicinal properties, including anti-inflammatory and analgesic effects, particularly in cancer-related pain management (Jin et al., 2014). Additionally, It is also used in veterinary medicine to promote digestion in livestock such as pigs, cattle, and sheep (Cao et al., 2020).

However, *Gelsemium* is highly toxic due to the presence of indole alkaloids such as gelsedine, gelsemine, humantenine, koumine, and especially gelsenicine, which is considered the most potent toxin (Huang et al., 2021; Sun et al., 2019). The entire plant—roots, stems, branches, and leaves—contains these compounds. Its extremely low toxic dose and rapid onset can lead to severe or fatal poisoning, particularly when misused or accidentally ingested. Experimental studies have shown that intraperitoneal administration of gelsenicine at doses >0.16 mg/kg in mice leads to acute poisoning symptoms within minutes, causing death in a dose-dependent manner. Neurological and respiratory depression are the primary clinical manifestations of its toxicity (Huang et al., 2025). These toxicological characteristics explain why accidental ingestion of *Gelsemium* can rapidly develop into severe foodborne poisoning events, especially in rural areas where wild plant foraging and traditional medicinal use remain common. Historically referenced in the Shennong Bencao Jing, *Gelsemium* gained the name “Duan-Chang-Cao” due to its lethal effects (Yang and Flaws, 1998). Today, most reported poisoning cases occur in southern China, particularly in Guangxi, Guangdong, and Fujian, with previous surveillance studies suggesting a relatively higher disease burden in Guangxi. In Guangxi, accidental ingestion remains the main cause, often linked to misidentification during foraging (Chen et al., 2020). The plant may be confused with edible or medicinal plants, including *Lonicera japonica* and other commonly collected herbs. In addition, the traditional practice of preparing homemade medicinal wine with wild plants may increase the risk of severe poisoning when *Gelsemium* is mistakenly used. From 2012 to 2017, *Gelsemium* was among the top three toxic plants causing death in Yunnan (Liu et al., 2018). A 2019 study identified Guangxi as the region with the highest incidence, where the consumption of medicinal wine containing *Gelsemium* accounted for many fatalities (Zhong et al., 2019a). In Hong Kong, surveillance data (2005–2017) showed most cases were due to misidentification or contamination (Chow et al., 2019).

International literature on *Gelsemium* poisoning is limited. Most case reports are in Chinese, and few studies examine long-term trends (Qu et al., 2021; Zhou et al., 2017). In Western countries, poisonings are rarer and often involve accidental ingestion by children (Froberg et al., 2007; Sriapha et al., 2015). In contrast, *Gelsemium* poisoning in southern China is more frequently associated with adult exposure, wild plant misidentification, and traditional medicinal use (Zhong et al., 2019b). Overall, global awareness and understanding of *Gelsemium* poisoning remain low.

Although interest in the medicinal value of *Gelsemium* continues, its potent toxicity poses an ongoing public health threat, especially in rural areas with limited access to emergency care. Systematic, long-term data on its epidemiological patterns, exposure characteristics, and clinical manifestations of *Gelsemium* poisoning at the provincial level remain scarce, particularly in Guangxi, where poisoning events continue to be reported. Therefore, this study aims to analyze surveillance data on *Gelsemium* poisoning reported in Guangxi from 2015 to 2024. By identifying key epidemiological and clinical characteristics, the findings are intended to inform targeted strategies for prevention, early detection, and effective management of *Gelsemium* poisoning in the region.

## 2 Methods

### 2.1 Study design and data sources

A secondary analysis was conducted using data from the Foodborne Disease outbreak Surveillance System of Guangxi Zhuang Autonomous Region, Southern China, covering the period from 1 January 2015 to 31 December 2024. Both the National Foodborne Disease Outbreak Surveillance System and the Guangxi regional system have been in operation since 2010 and 2012, respectively, aiming to monitor the burden of foodborne diseases. In Guangxi, 110 Centers for Disease Control and Prevention (CDCs) are responsible for reporting foodborne outbreaks after investigation. All outbreaks related to *Gelsemium* poisoning reported within this system during the study period were included in the analysis.

### 2.2 Data collection

#### 2.2.1 Case definition and data collection

According to the National Surveillance Protocol for Foodborne Diseases, a foodborne outbreak is defined as an incident in which patients experience diarrhea (≥3 episodes of abnormal stools), vomiting, or toxic symptoms suspected to be caused by food consumption. An outbreak is further characterized as an event in which two or more individuals develop similar clinical manifestations after consuming the same food within a similar time frame (Yang et al., 2025). CDC staff are required to investigate the source of the outbreak and report the complete event into the national surveillance system within 7 days. To confirm the causative agent in Gelsemium poisoning incidents, laboratory identification of Gelsemium toxins was performed using high-performance liquid chromatography–tandem mass spectrometry (LC-MS/MS) for the determination of 11 Gelsemium alkaloids, including gelsemine, in herbal wine or biological specimens. This validated method has been widely used in poisoning investigations and serves as a reference approach for emergency detection of Gelsemium poisoning events (Chen et al., 2023). For this secondary analysis, we included all Gelsemium-related foodborne outbreaks that occurred in Guangxi between 2015 and 2024. No sampling was performed; all reported incidents were included.

#### 2.2.2 Variables and variable definitions

The main outcome measure of this study was the occurrence of *Gelsemium* outbreak, characterized by the number of exposure cases, onset cases, hospitalizations, and deaths.

Independent variables included city, year, age group, season, cooking method, exposure place, plant source part, plant misidentification, and clinical symptoms. City was categorized into nine locations in Guangxi Province: Nanning, Chongzuo, Wuzhou, Yulin, Baise, Guigang, Hezhou, Qinzhou, and Laibin. The study period included the years 2015 through 2024. Age was grouped into four categories: 0–6, 7–19, 20–59, and ≥60 years. The season of outbreak occurrence was classified as spring (February–April), summer (May–July), autumn (August–October), or winter (November–January). Cooking methods were categorized as stewed soup/boiled water, alcohol infusion, fried dishes, and raw or freshly juiced preparations. Exposure places were recorded as household, rural banquet, or other. As the entire *Gelsemium* plant is toxic, the part consumed was categorized as rhizome, leaves, or pollen. In addition, we documented cases in which *Gelsemium* was misidentified as one of 15 morphologically similar plant species, in accordance with the National Protocol. Clinical symptoms were recorded and analyzed based on their frequency among reported cases.

#### 2.2.3 Data management and statistical analysis

Data from the Foodborne Disease Surveillance System were retrieved and analyzed using R software version 4.1.2 (R Foundation for Statistical Computing, 2020, Vienna, Austria). All reported *Gelsemium* poisoning events in Guangxi between 2015 and 2024 were included in this analysis. Key variables, including the number of cases, dates, locations, and outcomes, were complete for all included records. As no missing values were identified in these variables, no data imputation procedures were performed. No geographic distribution maps were generated in this study; therefore, geographic coordinate processing and issues related to mapping regulations were not applicable.

Epidemiological methods were applied to characterize events reported between 2015 and 2024 that met the criteria for plant-derived food poisoning. Descriptive statistics were used to summarize the results, with categorical data presented as frequencies and percentages. Incidence rates were calculated by dividing the number of onset cases by the number of exposure cases. Hospitalization rates were calculated by dividing the number of hospitalized cases by the number of onset cases, and fatality rates were calculated by dividing the number of deaths by the number of onset cases. Differences in incidence, hospitalization, and fatality rates across independent variables were assessed using the chi-square test. A two-sided p-value of less than 0.05 was considered statistically significant.

Joinpoint regression analysis was performed to examine temporal trends in incident case rates, incidence rates, and case fatality rates from 2015 to 2024 using the Joinpoint Regression Program (version 5.2.0.0). For years with zero observed cases, a value of 0.01 was added to the dependent variable to facilitate log transformation. The annual percent change (APC) and average annual percent change (AAPC) were calculated with 95% confidence intervals. A two-sided p-value < 0.05 was considered statistically significant.

## 3 Results

From 2015 to 2024, a total of 32 *Gelsemium* poisoning incidents were reported in Guangxi. The death rate was highest in the second quarter of 2015 (100%), followed by a high rate in the first quarter of 2018. The lowest death rate occurred in 2021, with no deaths reported. Joinpoint regression analysis showed that the incidence rate increased significantly from 2015 to 2017 (APC: 30.74%, *P* = 0.018), remained stable from 2017 to 2022 (APC: −0.72%, *P* = 0.384), and then decreased significantly from 2022 to 2024 (APC: −22.58%, *P* = 0.020). The overall AAPC from 2015 to 2024 was −0.13% (*P* = 0.911), indicating no significant long-term trend. For the case fatality rate, a significant sharp increase was observed from 2021 to 2024 (APC: 410.46%, *P* = 0.047), although this should be interpreted with caution due to the small number of deaths. Most incidents led to a high proportion of symptomatic cases and nearly all resulted in hospitalizations, with fluctuating fatality rates across the study period (Table 1).

**TABLE 1:** Joinpoint regression analysis of Gelsemium poisoning in Guangxi, 2015–2024.

Variables and segments	Year	APC (95% CI)	t	P-value
Incident cases rate				
Trend1	2015–2017	−16.17 (-50.98.43.34)	−1.41	0.293
Trend2	2017–2020	36.13 (-17.01,123.31)	2.68	0.115
Trend3	2020–2024	6.55 (-5.22.19.79)	2.33	0.145
AAPC (95% CI)	2015–2024	9.62 (-0.37.20.63)	1.88	0.060
Incidence rate				
Trend1	2015–2017	30.74 (11.81.52.87)	7.37	0.018
Trend2	2017–2022	−0.72 (-3.48.2.12)	−1.11	0.384
Trend3	2022–2024	−22.58 (-33.79,-9.45)	−7.04	0.020
AAPC (95% CI)	2015–2024	−0.13 (-2.45.2.24)	−0.11	0.911
Case fatality rate				
Trend1	2015–2018	6.20 (-78.17,416.54)	0.164	0.885
Trend2	2018–2021	−82.47 (-99.66,800.98)	−1.90	0.198
Trend3	2021–2024	410.46 (4.95,2382.82)	4.43	0.047
AAPC (95% CI)	2015–2024	−1.68 (-50.58.95.61)	−0.05	0.962

**FIGURE 1:**
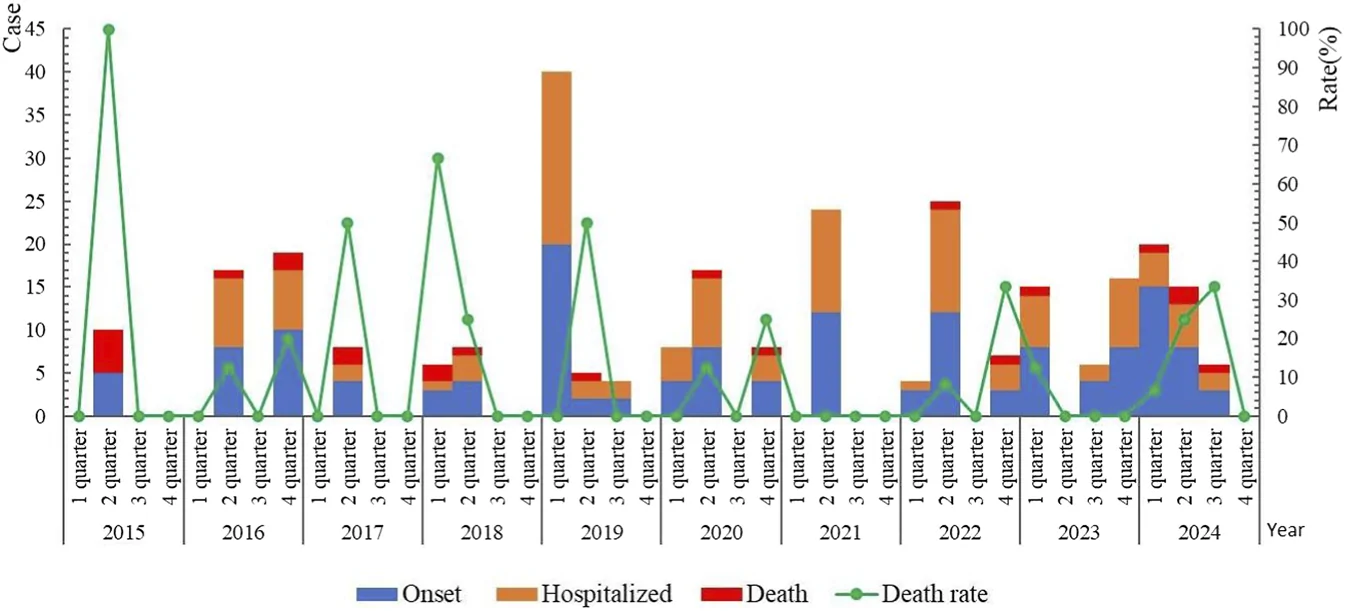
Trends in onset cases, hospitalizations, deaths, and case fatality rates of Gelsemium poisoning.

The fatality rate reached its highest level in the second quarter of 2015, at 100%, followed by another notable peak in the first quarter of 2018. In contrast, no deaths were reported in 2021, marking the lowest fatality rate during the surveillance period. However, the rate began to rise again after 2021, and by 2024, deaths were reported in three of the four-quarters, indicating a concerning upward trend. The number of onset cases peaked in the first quarter of 2019, with similarly high case counts reported in 2022, 2023, and 2024. Overall, onset trends showed substantial fluctuation throughout the decade. Hospitalizations mirrored these patterns, peaking in the first quarter of 2019, followed by elevated hospitalization numbers in 2021 and the second quarter of 2022. Regarding deaths, the second quarter of 2015 reported the highest number of fatalities, which then gradually declined through 2021. However, deaths subsequently increased again, culminating in a multi-quarter spike in 2024 (Figure 1).

The regional distribution of *Gelsemium* poisoning incidents is presented in Table 2. The incidence rate following *Gelsemium* consumption was 100% in Chongzuo, Yulin, Guigang, Qinzhou, and Laibin. With the exception of Baise, which exhibited a relatively lower incidence rate, other regions also showed high levels of exposure-related illness. A significant difference in incidence rates was observed among different cities (*P* < 0.001). The highest hospitalization rate occurred in Guigang (88.9%), followed closely by Yulin (87.0%). Wuzhou reported the highest fatality rate (42.1%), followed by Laibin (25.0%). Across the surveillance period from 2015 to 2024, most years reported an incidence rate of 100%, indicating the high pathogenic potential of *Gelsemium* exposure. Hospitalization rates peaked in 2019 and 2021, both reaching 100%. The fatality rate was highest in 2015 (100%) and lowest in 2021 (0%). Annual differences in incidence, hospitalization, and fatality rates were all statistically significant (*P* < 0.001). All age groups were affected by *Gelsemium* poisoning, with consistently high hospitalization rates across all four categories. The 20–59 age group exhibited the highest fatality rate (19.0%), suggesting greater vulnerability or possibly higher exposure levels in the working-age population. Seasonal analysis revealed the highest incidence in autumn (100%), followed by winter (97.1%), with significant variation in incidence between seasons (*P* < 0.001).

**TABLE 2:** Basic characteristic information of incidents.

Type	Sub-type	Outbreak	Exposure cases	Onset (rate)		Hospitalization (rate)		Death (rate)	
				Case	P	Case	P	Case	P
City	Nanning	8	35	33 (94.3)	<0.001	28 (84.8)	0.068	4 (12.1)	0.055
	Chongzuo	4	20	20 (100.0)		16 (80.0)		2 (10.0)	
	Wuzhou	4	24	19 (79.2)		10 (52.6)		8 (42.1)	
	Yulin	4	23	23 (100)		20 (87.0)		1 (4.3)	
	Baise	4	49	18 (36.7)		13 (72.2)		3 (16.7)	
	Guigang	3	18	18 (100.0)		16 (88.9)		1 (5.6)	
	Hezhou	2	18	10 (90.9)		7 (70.0)		2 (20.0)	
	Qinzhou	2	5	5 (100.0)		2 (40.0)		1 (20.0)	
	Laibin	1	4	4 (100.0)		3 (75.5)		1 (25.0)	
Year	2015	1	10	5 (50.0)	<0.001	0 (0.0)	<0.001	5 (100.0)	<0.001
	2016	4	19	18 (94.7)		15 (83.3)		3 (16.7)	
	2017	1	4	4 (100.0)		2 (50.0)		2 (50.0)	
	2018	2	7	7 (100.0)		4 (57.1)		3 (42.9)	
	2019	4	25	24 (96.0)		24 (100.0)		1 (4.2)	
	2020	4	16	16 (100.0)		15 (93.8)		2 (12.5)	
	2021	1	12	12 (100.0)		12 (100.0)		0 (0.0)	
	2022	4	20	18 (90.0)		16 (88.9)		2 (11.1)	
	2023	4	20	20 (100.0)		16 (80.0)		1 (5.0)	
	2024	7	56	26 (46.4)		11 (42.3)		4 (15.4)	
Age	0–6	-	-	5	—	5 (100.0)	0.058	0 (0.0)	0.175
	7–19	-	-	15		15 (100.0)		0 (0.0)	
	20–59	-	-	105		78 (74.3)		20 (19.0)	
	≥60	-	-	25		17 (68.0)		3 (12.0)	
Season	Autumn	5	21	21 (100.0)	<0.001	18 (85.7)	0.281	2 (9.5)	0.011
	Spring	15	102	71 (69.6)		55 (77.5)		9 (12.7)	
	Summer	7	31	24 (77.4)		15 (62.5)		9 (37.5)	
	Winter	5	35	34 (97.1)		27 (79.4)		3 (8.8)	

As shown in Table 3, the method of cooking significantly influenced the clinical severity of *Gelsemium* poisoning. Fried *Gelsemium* dishes were associated with both the highest incidence and hospitalization rates (100%), suggesting that heat processing may not sufficiently neutralize the plant’s toxic components. Poisoning through alcohol infusion was particularly lethal, with a fatality rate of 29.5%, the highest among all preparation methods. Exposure settings also played a critical role in the outcome of poisoning incidents. Both household and rural banquet exposures led to high incidence rates, but fatalities were significantly more common in household settings (21.1%). This highlights the urgent need for public health education targeting home-prepared herbal or wild plant-based meals, especially in regions where foraging is culturally embedded. The toxic components of *Gelsemium* are present throughout the plant, but consumption of different parts resulted in varying clinical outcomes. Leaves were the most commonly ingested part and were associated with the highest incidence (100%) and hospitalization rates (98.0%). However, ingestion of the rhizome led to the highest fatality rate (21.1%), followed closely by pollen (20.0%).

**TABLE 3:** The exposure of *Gelsemium* poisoning incidents in Guangxi.

Type	Sub-type	Outbreak	Exposure cases	Onset (rate)		Hospitalization (rate)		Death (rate)	
				Case	P	Case	P	Case	P
Cooking method	Stewed soup/Boiled water	14	68	67 (98.5)	<0.001	57 (85.1)	<0.001	7 (10.4)	0.005
	Infused alcohol	10	81	44 (54.3)		25 (56.8)		13 (29.5)	
	Fried dishes	4	22	22 (100.0)		22 (100)		0 (0.0)	
	Raw/Freshly juiced	4	18	17 (94.4)		11 (64.7)		3 (17.6)	
Exposure place	Household	27	154	115 (74.7)	0.004	86 (74.8)	0.013	21 (18.3)	0.109
	Rural banquet	2	20	20 (100.0)		20 (100)		0 (0.0)	
	Others	3	15	15 (100.0)		9 (60)		2 (13.3)	
Source part	Rhizome	22	128	90 (70.3)	<0.001	59 (65.6)	<0.001	19 (21.1)	0.024
	Leaves	8	50	50 (100.0)		49 (98)		2 (4.0)	
	Pollen	2	11	10 (90.9)		7 (70.0)		2 (20.0)	
Misrecognizes for the plant or foods	Unknow foods/Herbal	11	81	50 (61.7)	<0.001	38 (76.0)	<0.001	8 (16.0)	<0.001
	*Ficus hirta*	4	22	22 (100.0)		18 (81.8)		1 (4.5)	
	*Contaminated honey*	2	11	10 (90.9)		7 (70.0)		2 (20.0)	
	*Kadsura heteroclita*	2	6	6 (100.0)		1 (16.7)		2 (33.3)	
	*Lonicera japonica*	2	9	9 (100.0)		9 (100)		1 (11.1)	
	*Stephania tetrandra*	2	7	5 (71.4)		5 (100)		2 (40.0)	
	*Celastrus orbiculatus*	1	3	3 (100.0)		0 (0)		0 (0.0)	
	*Clematis chinensis*	1	16	16 (100.0)		16 (100)		0 (0.0)	
	*Cyclea barbata*	1	2	2 (100.0)		2 (100)		0 (0.0)	
	*Gracilaria spp*	1	9	9 (100.0)		9 (100)		0 (0.0)	
	*Millettia speciosa*	1	4	4 (100.0)		2 (50)		0 (0.0)	
	*Pachygone ovata*	1	10	5 (50.0)		0 (0)		5 (100.0)	
	*Senecio scandens*	1	2	2 (100.0)		2 (100)		1 (50.0)	
	*Smilax glabra*	1	3	3 (100.0)		3 (100)		0 (0.0)	
	*Uncaria rhynchophylla*	1	4	4 (100.0)		3 (75)		1 (25.0)	

Fifteen plant species were identified as commonly mistaken for *Gelsemium*, leading to poisoning incidents (Table 3). Among them, misidentifications involving *Ficus hirta*, *Kadsura heteroclita*, *L. japonica*, *Celastrus orbiculatus*, *Clematis chinensis*, *Cyclea barbata*, *Gracilaria spp*., *Millettia speciosa*, *Senecio scandens*, *Smilax glabra*, and *Uncaria rhynchophylla* were associated with a 100% incidence rate. The differences in incidence among various misidentified plants were statistically significant (*P* < 0.001). Hospitalization rates also reached 100% in cases involving *Lonicera japonica*, *Stephania tetrandra*, *Clematis chinensis*, *Cyclea barbata*, *Gracilaria spp.*, *Senecio scandens*, and *Smilax glabra*, with significant differences across misidentifications (*P* < 0.001). The most severe outcome was observed in cases where *Pachygone ovata* was mistaken for *Gelsemium*, resulting in a fatality rate of 100%. Overall, fatality rates varied significantly across different misidentified species (*P* < 0.001).

Clinically, the most frequently reported symptom was dizziness (69.8%), followed by blurred vision (57.3%), vomit (38.0%), headache (36.0%), and nausea (28.7%). Neurological symptoms and ocular signs were predominant, with blurred vision and bilateral ptosis being the most common ocular manifestations (Table 4).

**TABLE 4:** The clinical symptoms of cases.

Clinical symptoms	Frequency (n = 150)	Percentage (%)
Dizziness	132	69.8
Blurred vision	86	57.3
Vomit	57	38.0
Headache	54	36.0
Nausea	43	28.7
Coma	32	21.3
Dyspnoea	23	15.3
Cyanosis	12	8.0
Bilateral ptosis	10	6.7
Bellyache	6	4.0
Convulsions	6	4.0
Numbness (Lips, tongue, and fingertips)	6	4.0
Sensory numbness	2	1.3

## 4 Discussion

This study provides a comprehensive analysis of *Gelsemium* poisoning outbreaks in Guangxi, China, based on a decade of surveillance data. Our findings highlight significant regional, seasonal, and clinical patterns of poisoning, with important implications for public health prevention and clinical management. Despite *Gelsemium*’s well-known toxicity, its ingestion—largely due to misidentification with edible herbs—continues to result in substantial morbidity and mortality, especially in rural communities. The majority of cases involved adults and occurred predominantly in autumn and winter. Moreover, certain cooking methods and plant parts were associated with higher risks of severe outcomes or death.

The observed seasonal peaks of *Gelsemium* poisoning in autumn and winter may be linked to local customs, particularly the consumption of homemade herbal wine and wild plant harvesting activities during autumn. In Guangxi, herbal wine is traditionally consumed during colder months to “warm the body,” while *Gelsemium* roots are easily mistaken for edible herbs such as Ficus hirta, thereby increasing the risk of accidental ingestion (Zhong et al., 2019b; Wen et al., 2018). Autumn also represents the peak season for wild herb collection in rural areas, during which *Gelsemium* is frequently misidentified and gathered (Zhong et al., 2019b; Hu et al., 2020). These behavioral factors provide a plausible explanation for the seasonal distribution observed in this study.

The highest number of cases reported in Wuzhou can be attributed to a combination of ecological and socioeconomic factors. Wuzhou experiences a subtropical monsoon climate and features extensive hilly and mountainous terrain, which coincides with the optimal distribution area of *Gelsemium* (Zhao et al., 2025). Furthermore, this region is characterized by high population density, high mobility of migrant workers, and a deeply rooted custom of consuming homemade herbal wine (Zhong et al., 2019b).

Clinically, the most common symptoms observed in our dataset were dizziness, blurred vision, nausea, and vomit—symptoms consistent with known toxic effects of *Gelsemium* alkaloids on the central nervous and visual systems (Zhang et al., 2007). In addition to dizziness, other common symptoms included blurred vision, dilated pupils, nausea and vomiting, fatigue, and dry mouth. These manifestations are consistent with the known mechanism of *Gelsemium* alkaloids, which act on the central and autonomic nervous systems (Zhao et al., 2025). Specifically, blurred vision and dilated pupils suggest involvement of the visual pathway, whereas nausea and vomiting are indicative of autonomic nervous system dysfunction. This symptom pattern is consistent with the pharmacodynamics of gelsemine and koumine, which act on glycine receptors and other neural pathways, resulting in neurotoxicity and visual impairment (Lara et al., 2016). The predominance of neurotoxic and ocular manifestations supports previous pharmacological findings and may aid clinicians in early recognition of *Gelsemium* poisoning. However, the lack of specific antidotes further complicates treatment and places greater emphasis on timely recognition, supportive care, and rapid referral to specialized centers.

One of the most notable findings in our study was the increased lethality of alcohol-infused *Gelsemium* preparations. Although such infusions are traditionally used in some rural areas for pain relief or tonic purposes, they pose substantial risks due to enhanced alkaloid extraction over time (Wen et al., 2018). Our results showed that fatal intoxication could occur rapidly after ingestion of these preparations, consistent with previous toxicological reports (Xiang et al., 2016). Studies have shown that the methanol and ethanol extracts of *Gelsemium* elegans significantly increase the concentration of gelsemine and koumine alkaloids, contributing to higher toxicity in alcoholic solutions (Luo et al., 2020). This finding underscores the danger of traditional herbal alcohol infusions and calls for targeted health education in rural populations where these practices are still prevalent.

Additionally, our analysis showed that while leaf ingestion was the most frequently reported exposure type, consumption of the rhizome and pollen resulted in significantly higher death rates. This aligns with previous studies indicating that alkaloid concentrations are particularly high in the root and reproductive parts of the plant (Jin et al., 2014; Chen et al., 2022). These findings suggest that although leaf ingestion is more common, rhizome and pollen exposures are more dangerous and potentially more toxic per unit. The consumption of pollen typically involved honey mixed with *Gelsemium* pollen. Bees collect nectar from *Gelsemium* plants, producing honey that contains toxins, which can lead to poisoning (Zheng et al., 2019). Future preventive efforts should include specific warnings about the rhizome and flowering parts, which may be misidentified or overlooked during foraging and cooking.

Accidental ingestion resulting from plant misidentification remains the most common cause of *Gelsemium* poisoning. Our findings revealed frequent confusion between *Gelsemium* and other edible or medicinal plants, such as *F. hirta*, *L. japonica*, and *P. ovata* (Chow et al., 2019). In particular, *P. ovata* poses a unique threat, as it may parasitize *Gelsemium* and absorb its toxic alkaloids, representing a hidden yet dangerous route of exposure (Cheung et al., 2018). The leaves of *P. ovata* are typically oval-shaped (Lin et al., 2024). Due to their similar leaf morphology, *P. ovata* and *Gelsemium* can be difficult to distinguish with the naked eye, leading to accidental misidentification during harvesting. Additionally, some misidentified species may exhibit overlapping toxicological symptoms, leading to diagnostic delays and poorer outcomes (Zhou et al., 2017). These findings highlight the urgent need for targeted public health messaging and the development of simple, accessible tools for plant identification, especially in regions where wild herb foraging is common.

Although *Gelsemium* is a highly toxic plant, its poisoning profile in China exhibits distinct differences compared to other countries. Our findings revealed that poisoning incidents in Guangxi predominantly affected adults, particularly those aged 20–59 years. In contrast, studies from Thailand and the United States show that children and adolescents represent the majority of plant-related poisonings. In Thailand, for instance, children under 13 accounted for the largest share of poisoning cases, with most incidents resulting from unintentional ingestion during play or unsupervised foraging (Sriapha et al., 2015). Similarly, in the United States, exposures were primarily observed among 16-year-olds and typically occurred at home (Enfield et al., 2018). Conversely, reports of *Gelsemium* poisoning are extremely rare in the United Kingdom (Zhou et al., 2017). These discrepancies underscore the influence of regional ecology, cultural practices, and behavioral patterns on poisoning risk. While accidental ingestion is a common cause globally, the context differs substantially: in China, it is often linked to traditional herb collection, cooking, or medicinal use among adults, particularly in rural settings (Zhong et al., 2019b).

The CFR observed in this study is consistent with previously reported trends. For example, the CFR for *Gelsemium* poisoning in Guangxi from 2015 to 2017 was 47.6% (Zhong et al., 2019b). Herbal wine ingestion was the predominant exposure route in Guangxi, with a CFR of 77.8% (Zhong et al., 2019b). Similarly, a case report from Heyuan, Guangdong, documented self-brewed herbal wine as the exposure route (Wen et al., 2018). These findings suggest that herbal wine ingestion is a common exposure pathway across South China, and prevention strategies should specifically target this high-risk behavior. Effective prevention strategies must also consider the local cultural context, including deeply rooted traditions of herbal medicine and homemade herbal wine consumption. Regional differences in CFR may be attributed to significant disparities in emergency response times (Shen, 2025), the unequal distribution of medical resources (e.g., low density of emergency stations and shortage of professional personnel in rural areas) (Li et al., 2022), as well as variations in plant toxicity. Notably, the alkaloid profiles of *Gelsemium* differ significantly across different plant organs and growth stages, with the gelsenicine type being the predominant toxic component (Wu et al., 2022).

The significant decline in *Gelsemium* poisoning incidence observed during 2022–2024 in this study is consistent with the changing epidemiological trends of poisoning reported globally and in some regions during the COVID-19 pandemic. Tan et al. (Tan et al., 2022) reported a marked decrease in poisoning cases due to contact with toxic plants and animals in Taiwan during the stay-at-home period in 2020, attributing this reduction to decreased outdoor and industrial activities. Similarly, a medical center in Taiwan documented a notable decline in emergency department visits for poisoning during the pandemic (Chen et al., 2025). Within mainland China, a multicenter study in Jiangsu Province also confirmed that COVID-19 substantially altered the epidemiological profile of acute poisoning (Wang et al., 2025). The significant declining trend observed during 2022–2024 may be associated with the strict population movement restrictions implemented during the pandemic, as well as the cumulative effects of preceding health education campaigns aimed at improving awareness of plant toxicity.

Finally, given that family banquets and rural gatherings were the most common exposure settings, a finding consistent with prior research (He et al., 2022; Ma et al., 2022). These should be prioritized for preventive interventions. Improper foraging and unsafe preparation practices during such communal events often lead to larger clusters of affected individuals. Strengthening food safety supervision in these contexts, along with educational campaigns focused on plant toxicity awareness, could substantially reduce the incidence of *Gelsemium* poisoning. Additionally, future research should explore rapid diagnostic techniques and candidate antidotes for alkaloid reversal, while establishing regional poison control networks to support frontline response. However, implementing regional poison control networks still faces logistical challenges, including limited medical resources in rural areas, insufficient capacity for toxic plant identification at the grassroots level, and high demands for funding and specialized personnel. Future research should prioritize the development of specific antidotes to neutralize *Gelsemium* alkaloid toxicity. In addition, rapid field detection methods—such as immunochromatographic test strips or portable biosensors—should be developed to assist medical personnel in remote areas with early diagnosis and timely treatment. Such tools would be particularly valuable in resource-limited rural settings.

Few limitations were found in our study. As a secondary analysis of surveillance data, underreporting and data quality inconsistencies are possible. Toxicological confirmation was not available for all cases, and diagnoses relied on clinical and epidemiological investigation. Despite these limitations, this study provides the most extensive overview to date of *Gelsemium*-related outbreaks in southern China. Although this study focuses on Guangxi, the methodology and prevention strategies—including seasonal health education, plant identification tools, and regional poison control networks—may serve as a reference for other Southeast Asian regions with similar flora. Given that *Gelsemium* is distributed across multiple countries in this region, the lessons learned from Guangxi could have broader applicability.

## 5 Conclusion

The epidemiology and clinical characteristics of *Gelsemium* poisoning in Guangxi, Southern China, reveal a high prevalence and mortality rate, largely attributable to accidental misidentification of the toxic herb. Most poisoning events occur during household and rural banquet settings, with neurological symptoms—especially dizziness and visual disturbances—being the predominant clinical manifestations. These findings provide a solid evidence base for enhancing public health interventions. Strengthening food safety regulations and delivering targeted education on poisonous plant identification to high-risk populations are essential. Additionally, public advisories during seasonal peaks should stress avoiding contact with or harvesting of unidentified wild plants. Such measures are critical to timely and effective prevention and control of *Gelsemium* poisoning in Guangxi and similar endemic regions.

## Data Availability

The data analyzed in this study is subject to the following licenses/restrictions: the dataset originates from routine public health surveillance and outbreak investigations and is subject to institutional and data protection restrictions. Therefore, it is not publicly available but may be accessed upon reasonable request and with approval from the Guangxi Zhuang Autonomous Region Centre for Disease Control and Prevention. Requests to access these datasets should be directed to Yanxu Zhong, gxcdczyx@163.com.
