# Correction: Epidemiological and clinical analysis of Gelsemium poisoning in Guangxi, Southern China: a decade of surveillance data

**DOI:** 10.3389/ftox.2026.1974873

**Published:** 2026-09-01

**Authors:** Yanxu Zhong, Mengmeng Shi, Wei Mao, Huitian Deng, Xiaofeng Huang, Yuyan Jiang

**Affiliations:** 1 Guangxi Zhuang Autonomous Region Centre for Disease Control and Prevention, Nanning, China; 2 Guangxi Key Laboratory for the Prevention and Control of Viral Hepatitis, Guangxi Zhuang Autonomous Region Centre for Disease Control and Prevention, Nanning, China; 3 Guangxi Zhuang Autonomous Region Academy of Preventive Medicine, Nanning, China; 4 School of Public Health, Guangxi Medical University, Nanning, China; 5 School of Public Health, Youjiang Medical University for Nationalities, Baise, China

**Keywords:** clinical features, epidemiology, gelsemium, poisoning, surveillance

An incorrect grant number was provided for the Guangxi Natural Science Foundation. The incorrect grant number was **“2025JJA140370”**. The correct grant number is **“2026GXNSFAA00641330”**.

The original version of this article has been updated.

